# Role of endogenous nitric oxide on PAF-induced vascular and respiratory effects

**DOI:** 10.1155/S0962935195000214

**Published:** 1995-03

**Authors:** M. Clement, M. Albertini

**Affiliations:** 1 Institute of Veterinary Physiology and Biochemistry University of Milan Via Celoria 10 Milan 20133 Italy

## Abstract

The role of endogenous nitric oxide (NO) on vascular and respiratory smooth muscle basal tone was evaluated in six anaesthetized, paralysed, mechanically ventilated pigs. The involvement of endogenous NO in PAF-induced shock and airway hyperresponsiveness was also studied. PAF (50 ng/kg, i.v.) was administered before and after pretreatment with N^G^-nitro-L-arginine methyl ester (L-NAME, 10 mg/kg, i.v.), an NO synthesis inhibitor. PAF was also administered to three of these pigs after indomethacin infusion (3 mg/kg, i.v.). In normal pigs, L-NAME increased systemic and pulmonary vascular resistances, caused pulmonary hypertension and reduced cardiac output and stroke volume. The pulmonary vascular responses were correlated with the increase in static and dynamic lung elastances, without changing lung resistance. Inhibition of NO synthesis enhanced the PAF-dependent increase in total, intrinsic and viscoelastic lung resistances, without affecting lung elastances or cardiac activity. The systemic hypotensive effect of PAF was not abolished by pretreatment with L-NAME or indomethacin. This indicates that systemic hypotension is not correlated with the release of endogenous NO or prostacyclines. Indomethacin completely abolished the PAF-dependent respiratory effects.


Mediators of Inflammation 4, 124–129 (1995)

### Role of endogenous nitric oxide on PAF-induced vascular and respiratory effects

M. Clement and M. Albertini[CA]

Institute of Veterinary Physiology and Biochemistry, University of Milan, Via Celoria 10, 20133 Milan, Italy

[CA] Corresponding Author

**The role of endogenous nitric oxide (NO) on vascular and respiratory smooth muscle basal tone was evaluated in six anaesthetized, paralysed, mechanically ventilated pigs. The involvement of endogenous NO in PAF-induced shock and airway hyperresponsiveness was also studied. PAF (50 ng/kg, i.v.) was administered before and after pretreatment with $N^G$-nitro-L-arginine methyl ester (L-NAME, 10 mg/kg, i.v.), an NO synthesis inhibitor. PAF was also administered to three of these pigs after indomethacin infusion (3 mg/kg, i.v.). In normal pigs, L-NAME increased systemic and pulmonary vascular resistances, caused pulmonary hypertension and reduced cardiac output and stroke volume. The pulmonary vascular responses were correlated with the increase in static and dynamic lung elastances, without changing lung resistance. Inhibition of NO synthesis enhanced the PAF-dependent increase in total, intrinsic and viscoelastic lung resistances, without affecting lung elastances or cardiac activity. The systemic hypotensive effect of PAF was not abolished by pretreatment with L-NAME or indomethacin. This indicates that systemic hypotension is not correlated with the release of endogenous NO or prostacyclines. Indomethacin completely abolished the PAF-dependent respiratory effects.**

**Key words:** Nitric oxide, Platelet activating factor, Respiratory resistance, Vascular tone

### Introduction

It is known that endothelial release of nitric oxide (NO) acts as a physiological regulator of vascular tone and blood pressure.[1–3] Because NO is also produced by several types of pulmonary cells,[4,5] it may be found in respiratory bronchioli and alveoli. The increased production of NO, found in exhaled air of asthmatic patients,[4] reflects the role of endogenous nitric oxide as a modulator of bronchial tone.[6,7] Consequently, airway epithelium plays the same role as endothelium in regulating the tone of underlying smooth muscle. Recently, a modulatory effect of epithelium on the contractile responses of airway smooth muscle to spasmogens[7] has been described. Airway epithelium plays this critical role in the regulation of airway functions[7] producing not only nitric oxide, but also a large number of inflammatory mediators, including prostaglandins, endothelin-1, and various cytokines. By releasing NO, the epithelial layer acts as a protective barrier against spasmogenic agents that are released in inflammatory disorders.

As suggested by Nijkamp *et al.*,[8] diminished production of NO after epithelial damage, typical of asthma, contributes to airway hyperresponsiveness,[9] which may be due to a marked release of platelet activating factor (PAF), a typical inflammatory and allergic mediator of asthma.[10] PAF is a potent bronchoconstrictor, and also induces strong pulmonary hypertension and systemic hypotension. Moritoki *et al.*[11] have suggested that this systemic hypotension can be due to production of nitric oxide.

The aim of this present study was to evaluate the role of endogenous NO on circulatory and respiratory systems and its involvement in PAF-induced shock and bronchoconstriction. To evaluate the role of endogenous NO in the normal pig or during PAF-dependent shock, we used $N^G$-nitro-L-arginine methyl ester (L-NAME), an arginine analogue, which acts as an inhibitor of NO synthesis.

### Materials and Methods

Six Large White pigs, of either sex, weighing 21.9 ± 1.3 (S.E.M.) kg, were used. The animals, sedated with 1% propionylpromazine hydrochloride (0.05 ml/kg, i.m.), were anaesthetized with 15 mg/kg thiopental-sodium injected into the auricular vein. The depth of anaesthesia was maintained by infusion, drop by drop, of thiopental-sodium (9 mg/kg/h). The animals, tied in the supine position on a heated operating table, were tracheostomized, paralysed with pancuronium bromide (0.02 mg/kg, i.v.) and mechanically ventilated (Servoventilator Sie-

© 1995 Rapid Communications of Oxford Ltd mens 900 C). When necessary, additional paralysing drug was administered during the experiments.

An endotracheal tube was inserted into the lower portion of the extrathoracic trachea. A side port of the tracheal cannula was connected to a pressure transducer (Statham 15299, Gould, Hatorey, Puerto Rico) for measurement of tracheal pressure. A heated pneumotachograph (Fleisch no 2, Fleisch Lausanne, Switzerland) was connected to the proximal end of the endotracheal tube and to the 'Y' piece of the ventilator to evaluate respiratory flow. Electronic integration of the flow signal gave the tidal volume. The pressure drop across the two ports of the pneumotachograph was measured with a differential pressure transducer (Statham PM15, 10846). The response of the pneumotachograph was linear over the experimental range of flows. To reduce the effects of the compliance of the system on the mechanical measurements, a fixed length standard low-compliance tube was used (2 cm i.d. 60 cm long) to connect the animals to the ventilator. The equipment's flow resistance was 0.5 $cmH_2O$/l/s and the equipment's dead space was 29.5 ml. The intrapleural pressure was evaluated by an air-filled polyethylene catheter tied into the lower 7th right intercostal space. Transpulmonary pressure ($P_L$) was measured as the difference between tracheal and intrapleural pressure, using a differential pressure transducer (Validyne MP 45, ± 100 $cmH_2O$; Validyne Inc., Northridge, CA, USA). Special care was taken to avoid air leaks from the tracheal and pleural cannulae and breathing circuit.

A balloon-tipped catheter (Swan Ganz 5F) was introduced into the left brachial vein and allowed to float through the right heart to the pulmonary artery. Polyethylene catheters were inserted into the right femoral artery to record blood pressure and into the right femoral vein for drug administration. Systemic and pulmonary arterial pressure were recorded by connecting the catheters to a fluid-filled capacitance manometer (Bell & Howell 4-422). Cardiac output (CO) was evaluated by the thermodilution method (Cardiac Output Computer 701 IL). All parameters were calibrated independently and recorded simultaneously on a multichannel pen recorder (model 8K40; NEC San-Ei instruments, Ltd, Tokyo, Japan). For subsequent analysis of data $P_L$ and flow were also recorded on an FM magnetic tape recorder (Racal Store 4, Racal Recorders Ltd, Southampton, UK). The signals were played back to an Olivetti 486 (Ivrea, Italy) personal computer by a 12-bit analogue-to-digital board at a sample frequency of 2000 Hz. Heart rate, mean arterial pressure (MAP) and mean pulmonary arterial pressure (MPAP) were evaluated from the polygraph tracings. Stroke volume (SV) and systemic (SVR) and pulmonary (PVR) vascular resistances were calculated with standard formulae.

*Procedure and data analysis:* The baseline ventilator settings were a fixed inflation volume of 0.2 ± 0.01 (S.E.M.) l and a fixed inspiratory flow of 0.25 ± 0.01 (S.E.M.) l/s. Respiratory frequency was 23 ± 2 (S.E.M.) breaths/min and the ratio of inspiratory time to total breathing cycle duration was 0.33 ± 0.01 (S.E.M.). Respiratory mechanics values were assessed by the constant flow inspiratory occlusion method.[12,13] For each breath, airway occlusion was followed by a rapid initial drop in transpulmonary pressure ($P_1$) and was maintained until an apparent plateau ($P_2$), representing the end-inspiratory elastic recoil pressure, was reached (5–6 s). The difference between the peak of transpulmonary pressure ($P_{max}$) and plateau pressure, divided by the immediately preceding steady flow provided the total lung resistance ($R_{max,L}$). The initial pressure drop in $P_L$ from $P_{max}$ to $P_1$ divided by the immediately preceding steady flow provides the intrinsic resistance of lung ($R_{int,L}$). Dividing $P_1$–$P_2$ by the pre-occlusion flow we obtained the additional effective resistance ($\Delta$RL) due to the viscoelastic properties of the thoracic tissues and the time constant inequalities within the lung.[14] The static ($E_{st,L}$) and dynamic ($E_{dyn,L}$) elastances of the lung were obtained by dividing $P_2$ or $P_1$ by the inspiratory volume.

*Protocol:* After evaluation of control values, PAF freshly mixed in normal saline solution was administered i.v. at a dose of 50 ng/kg.[15] When baseline values had recovered (about 45 min), L-NAME was administered through the femoral vein at a dose of 10 mg/kg. Preliminary studies showed that this dose causes vascular effects within 10 min and that these are stable for at least 120 min. Thirty minutes after L-NAME pretreatment, PAF was again administered at the same dose. After recovery from PAF effects, indomethacin was also administered to three of six pigs pretreated with L-NAME, through the femoral vein, at a dose of 3 mg/kg. The pigs were treated again with PAF 30 min after indomethacin administration.

*Data analysis and statistics:* Results are expressed as means ± S.E.M. The significance of differences between two sets of data was assessed by the two-tailed *t*-test for paired data. A significant difference was defined as $p < 0.05$.

### Results

The circulatory parameters, evaluated under all experimental conditions, are given in Table 1. PAF caused a prompt and short-lasting decrease in mean arterial pressure and a marked increase in mean pulmonary arterial pressure. These haemodynamic changes reached a peak in 10–20 s and were associated with transient cardiac arrest. While MAP rapidly returned to control values, pulmonary hypertension lasted 20 min. The vascular changes were associated

**Table 1.** Circulatory parameters in all experimental conditions

| | *n* | MAP (mmHg) | MPAP (mmHg) | SV (ml) | CO (l/min) | PVR (mmHg/l/m) | SVR (mmHg/l/m) |
|---|---|---|---|---|---|---|---|
| **Control** | | | | | | | |
| Mean | 6 | 106.25 | 22.42 | 28.63 | 2.59 | 8.93 | 42.50 |
| S.E.M. | | 4.54 | 1.68 | 2.33 | 0.24 | 0.79 | 2.92 |
| **PAF** | | | | | | | |
| Mean | 6 | 58.19 | 56.15 | 6.16 | 0.76 | 81.18 | 82.34 |
| S.E.M. | | 6.07 | 2.26 | 1.49 | 0.10 | 8.41 | 9.92 |
| *p* | | * | * | * | * | * | * |
| **Control** | | | | | | | |
| Mean | 6 | 94.11 | 22.82 | 25.05 | 2.13 | 11.11 | 46.94 |
| S.E.M. | | 4.27 | 2.35 | 2.81 | 0.20 | 1.02 | 5.30 |
| **L-NAME** | | | | | | | |
| Mean | 6 | 105.28 | 34.32 | 15.76 | 1.34 | 28.19 | 79.15 |
| S.E.M. | | 13.64 | 3.49 | 1.96 | 0.15 | 4.84 | 7.02 |
| *p* | | | * | * | * | * | * |
| **PAF** | | | | | | | |
| Mean | 6 | 44.72 | 47.50 | 6.15 | 0.78 | 84.62 | 82.29 |
| S.E.M. | | 3.51 | 2.21 | 2.65 | 0.20 | 21.83 | 22.79 |
| *p* | | ° | ° | ° | ° | ° | ° |

*n*, number of pigs; MAP, mean arterial pressure; MPAP, mean pulmonary arterial pressure; SV, stroke volume; CO, cardiac output; PVR, pulmonary vascular resistances; SVR, systemic vascular resistances. *$p < 0.05$ *vs* control conditions; °$p < 0.05$ *vs* L-NAME administration.

with significant decreases in stroke volume and cardiac output. PAF administration increased pulmonary and systemic vascular resistances, but the decrease in MAP was the cause of the lesser change in SVR.

L-NAME administration did not affect systemic arterial pressure, but significantly increased MPAP. L-NAME also strongly decreased CO, due to the reduction in SV, because the heart rate did not change (data not reported). The changes in cardiac activity were responsible for the rises in SVR and in PVR.

Pretreatment with L-NAME did not prevent either systemic hypotension or pulmonary hypertension caused by PAF administration. In fact, in this case too, PAF increased MPAP (147.72 ± 17.55%), even though this effect was less than that obtained before L-NAME (263.78 ± 32.24%, $p < 0.05$). The L-NAME-dependent decreases in stroke volume and cardiac output were strengthened by PAF, which, again, caused transient cardiac arrest. In pigs pretreated with L-NAME, the administration of PAF did not modify the SVR, but caused a further marked increase in PVR, to values similar to that observed when PAF was administered before L-NAME. In three of the six pigs pretreated with L-NAME, indomethacin administration did not prevent PAF-dependent systemic hypotension.

The changes in lung resistances are shown in Fig. 1. PAF administration caused a marked bronchoconstriction, all shown by the rise in $R_{int,L}$ and a large change in the viscoelastic properties of the lung, reflected by the increase in $\Delta RL$. Because $R_{max,L}$ is the sum of $R_{int,L}$ and $\Delta RL$, the increases in intrinsic and viscoelastic resistances were the cause of the rise in the total lung resistance. L-NAME administration did not affect the resistances of the lung, but did enhance the effects of PAF on $R_{int,L}$, $\Delta RL$ and $R_{max,L}$. Indomethacin, administered to pigs pretreated with L-NAME, did not affect pulmonary resistances and completely abolished the effects of PAF.

Figure 2 shows the changes in static (A) and dynamic (B) elastances of the lung under all experimental conditions. The results show that PAF administration significantly increased both static and dynamic elastances. Under control conditions L-NAME significantly increased $E_{st,L}$ and $E_{dyn,L}$, but did not alter the effects of PAF on static and dynamic elastances. In pigs pretreated with L-NAME, the PAF-dependent rise in $E_{st,L}$ and $E_{dyn,L}$ was similar to that observed without L-NAME. As for lung resistances, pretreatment with indomethacin associated with L-NAME prevented PAF-dependent changes in static and dynamic elastances.

Figure 3 shows the correlation between $E_{st,L}$ and MPAP. The increases in MPAP and $E_{st,L}$ caused by L-NAME or by PAF were similar. When PAF was administered after pretreatment with L-NAME, the lesser increase in MPAP was correlated with a greater increase in $E_{st,L}$.

### Discussion

This study shows that endogenous NO acts as a modulator not only of vascular tone, but also of bronchial tone. In addition, our data show that there

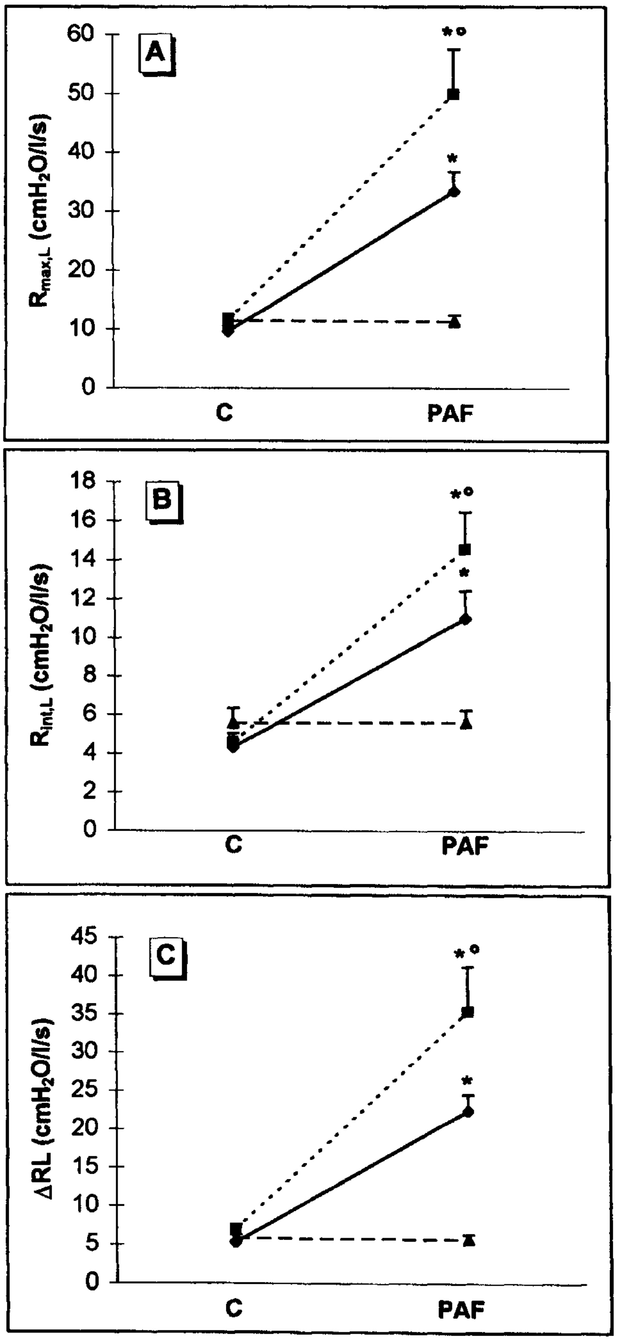

FIG. 1. Panel A, total lung resistance ($R_{max,L}$); Panel B, intrinsic lung resistance ($R_{int,L}$) and Panel C, viscoelastic lung resistance (ΔRL) under control conditions (C) and after PAF administered to normal pigs (——),), to pigs pretreated with L-NAME (........) and to pigs pretreated with L-NAME + indomethacin (- - -). Values are means ± S.E.M. $^*p < 0.05$ *vs* control conditions. $^\circ p < 0.05$ *vs* PAF administered under control conditions.

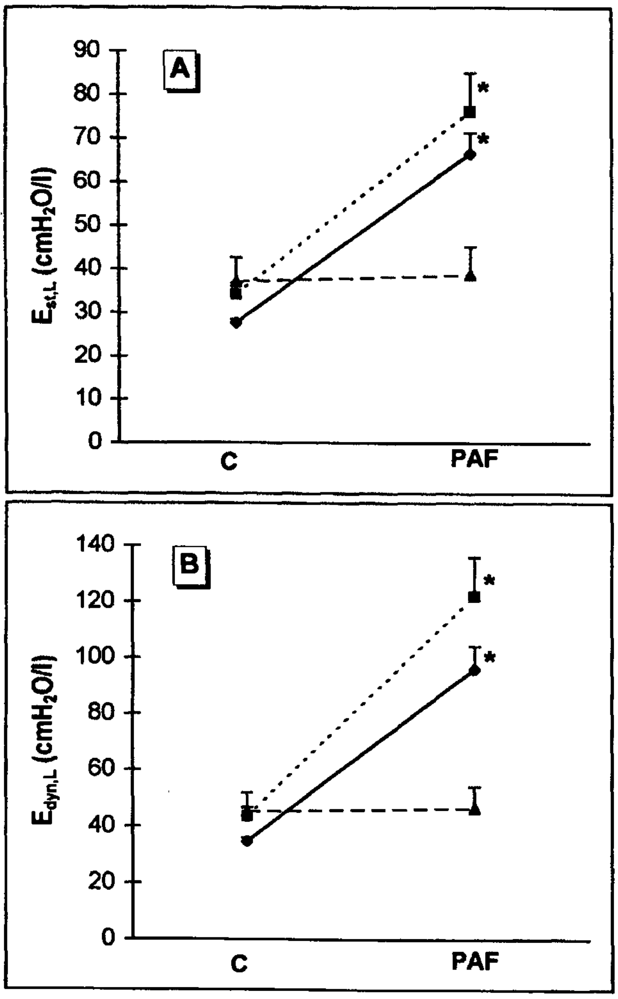

FIG. 2. Panel A; static lung elastance ($E_{st,L}$) and Panel B, dynamic lung elastance ($E_{dyn,L}$) under control conditions (C) and after PAF administered to normal pigs (——), to pigs pretreated with L-NAME (........) and to pigs pretreated with L-NAME + indomethacin (- - -). Values are means ± S.E.M. $^*p < 0.05$ *vs* control conditions.

are different NO-mediated regulatory mechanisms in vascular and respiratory smooth muscle, because bronchial tone is modulated by NO only when smooth muscles have been preconstricted by PAF. The block of NO synthesis by L-NAME increases pulmonary and systemic vascular resistances, reflecting the modulatory role of NO on vascular smooth muscle, but did not modify lung resistances. This lack of change reflects the basal bronchial tone in pig, which, unlike that in other species, is not affected by inhibition of NO synthesis.

Our data also seem to suggest that the sensitivity to NO differs in the pulmonary and systemic vascular beds. After L-NAME administration MPAP increased, while the systemic blood pressure did not. The increase in MPAP after block of endogenous NO confirms the data of Persson *et al.*[16] showing that pulmonary vascular tone is regulated by nitric oxide. Even though it is not possible to exclude a difference in NO sensitivity in the two vascular districts, it seems probable that the systemic vasoconstrictor effect is masked by the marked decrease in CO consequent to an impairment of cardiac activity. The latter effect may be caused by a sustained coronary vasoconstriction due to an inhibition of NO synthesis. It is known that NO is produced continuously by coronary endothelium[17] and, as suggested by Christie *et al.*,[18,19] the smooth muscle of pig coronary artery is extremely sensitive to its action. Consequently, a block of NO, causing coronary ischaemia, may be responsible for impairment of cardiac activity.

As suggested by Moritoki *et al.*,[11] the release of NO may be stimulated by PAF. Consequently, PAF ad-

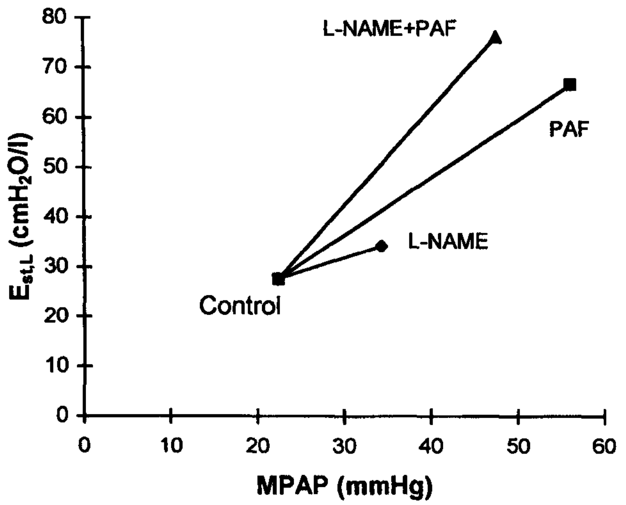

FIG. 3. Influence of mean pulmonary arterial pressure (MPAP) on static lung elastance ($E_{st,L}$) under all the experimental conditions.

ministration to pigs pretreated with L-NAME would induce a greater increase in MPAP than that observed without a block of endogenous NO. It is known that PAF causes the release of many vasoactive mediators, hence the lesser increase in MPAP we observed in pigs pretreated with L-NAME may reflect an imbalance between the effects of the vasoconstrictors and vasodilators. The major vasodilators are NO and $PGI_2$, which are released in parallel by endothelial cells.[20,21] The block of NO release alters the coupling between NO and $PGI_2$ probably enhancing $PGI_2$ activity. An imbalance between vasoconstrictors and vasodilators may be responsible also of PAF-dependent systemic hypotension. Takekoshi *et al.*[22] have suggested that the PAF-dependent systemic hypotension in the dog is due to a release of NO, while our results, confirming previous data,[23] show the permanence of systemic vasodilatation in pigs pretreated with L-NAME. This shows that in this species PAF-dependent systemic hypotension is not due to release of NO. $PGI_2$ might also be responsible for the PAF-dependent systemic hypotension, but the permanence of this hypotension even after pretreatment with indomethacin[23] shows that the prostacyclin is not involved in this mechanism. Hence the prompt and short-lasting PAF-dependent hypotensive effect associated with a marked decrease in CO accounts for the cardiac involvement.

When the effects of L-NAME on the respiratory system are evaluated, the different regulatory mechanisms of endogenous NO on the basal tones of pulmonary vascular and bronchial smooth muscle becomes obvious. Our results show that endogenous NO block does not affect respiratory resistances, only vascular resistances. However, Nijkamp *et al.*[8] who administered L-NAME by aerosol, suggested that endogenous NO has a role in the regulation of basal bronchial tone. The differences in response might be due to different methods of administration. Epithelial cells, as suggested by Nijkamp *et al.*,[8] probably contribute to NO synthesis and, hence, administration of L-NAME by aerosol may favour the side effects of NO inhibition on the epithelial lining of the airway smooth muscles. The difference between the intraluminal and extraluminal responses, confirming the hypothesis of Nijkamp *et al.*,[8] indicates the intrinsic role of airway epithelium as a modulator of bronchial tone. In pigs, the role of endogenous NO becomes evident during PAF-dependent bronchoconstriction. Our results show that the block of endogenous NO by L-NAME strengthened PAF's effects, further increasing the RL. It is known that PAF is a mediator in many pulmonary inflammatory and allergic disorders, such as asthma.[10] The epithelial layer in asthmatic patients is often damaged and the degree of this damage is associated with the degree of airway hyperresponsiveness.[9] This suggests that NO produced by airway epithelial cells is involved in the airway's defensive mechanisms and in the regulation of bronchial tone. The greater increase in $R_{max,L}$ observed when PAF was administered to pigs pretreated with L-NAME was due not only to a bronchoconstrictor effect, as shown by the increase in $R_{int,L}$, but essentially to an increase in ΔRL. The latter effect, reflecting the viscoelastic properties of the lung, is evidence of the parenchymal and vascular damage caused by PAF. The major effect is pulmonary hypertension, causing lung oedema. Thus, the smooth muscle contraction, associated with a marked pulmonary hypertension and interstitial oedema, causes the change in ΔRL and, consequently, in elastance, which seems to be particularly correlated with the change in MPAP (Fig. 3). The rise in MPAP due to L-NAME administration is correlated with a change in elastance even when respiratory resistance does not change. The greater increase in $E_{st,L}$, after PAF administration occurs in the presence of the greater increase in MPAP, and this may induce more oedema. When PAF was administered after endogenous NO had been blocked, MPAP increased less, but the change in $E_{st,L}$ was greater. Combined administration of L-NAME and PAF, as suggested by Filep *et al.*,[24] evokes a significant increase in albumin extravasation into the pulmonary parenchyma, and potentiates the vascular permeability effect of PAF. This may be the reason for the greater change in $E_{st,L}$ we observed even in the presence of a lower increase in MPAP, and may reflect the protective role of NO on the lung.

In conclusion, this study shows that NO is released by both endothelial and epithelial cells. Inhibition of NO synthesis by L-NAME administration increases the inflammatory cell accumulation induced by PAF.[25] Thus, nitric oxide may be considered an endogenous defensive mechanism against inflammatory, obstructive and hypertensive lung diseases, and may be an important factor in the regulation of airway hyperresponsiveness[9] and in the maintenance of bronchial tone.[26,27]

### References

1. Palmer RMJ, Ferrige AG, Moncada S. Nitric oxide release accounts for the biological activity of endothelium-derived relaxing factor. Nature 1987; **327**: 524–526.
2. Ignarro LJ, Byrns RE, Woods KS. Biochemical and pharmacological properties of endothelium-derived relaxing factor and its similarity to nitric oxide radical. In: Vanhoutte PM, ed. *Vasodilation: Vascular Smooth Muscle, Peptides, Autonomic Nerves and Endothelium.* New York Raven Press, 1988; 427–436.
3. Moncada S, Higgs A. The L-arginine–nitric oxide pathway. *New Engl J Med* 1993; **329**: 2002–2012.
4. Kharitonov SA, Yates D, Robbins RA, Logan-Sinclair R, Shinebourne EA, Barnes PJ. Increased nitric oxide in exhaled air of asthmatic patients. *Lancet* 1994; **343**: 133–135.
5. Barnes PJ. Nitric oxide and airways. *Eur Resp J* 1993; **6**: 163–165.
6. Persson MG, Wiklund NP, Gustafsson LE. Endogenous nitric oxide in single exhalation and the change during exercise. *Am Rev Respir Dis* 1993; **148**: 1210–1214.
7. Barnes PJ. Epithelium acts as a modulator and a diffusion barrier in the responses of canine airway smooth muscle. *J Appl Physiol* 1994 **76**: 1841–1842.
8. Nijkamp FP, Van Der Linde HJ, Folkerts G. Nitric oxide synthesis inhibitors induce airway hyperresponsiveness in the guinea pig *in vivo* and *in vitro*. Role of the epithelium. *Am Rev Respir Dis* 1993; **148**: 727–734.
9. Djukanovic R, Roche WR, Wilson JW, Beesley CRW, Twentyman OP, Howarth PH, Holgate ST. Mucosal inflammation in asthma. *Am Rev Respir Dis* 1990; **142**: 434–457.
10. Braquet P, Touqui L, Shen TY, Vargaftig BB. Perspectives in platelet-activating factor research. *Pharmacol Rev* 1987; **39**: 97–145.
11. Moritoki H, Hisayama T, Takeuchi S, Miyano H, Kondoh W. Involvement of nitric oxide pathway in the PAF-induced relaxation of rat thoracic aorta. *Br J Pharmacol* 1992; **107**: 196–201.
12. Bates JHT, Brown KA, Kochi T. Respiratory mechanics in the normal dog determined by expiratory flow interruption. *J Appl Physiol* 1989; **67**: 2276–2285.
13. Albertini M, Clement MG. Inhaled nitric oxide reverses vascular and respiratory effects of ET-1 and PAF in pigs. *Mediators of Inflamm* 1994; **3**: 439–444.
14. Bates JHT, Rossi A, Milic-Emili J. Analysis of the behaviour of the respiratory system with constant inspiratory flow. *J Appl Physiol* 1985; **58**: 1840–1848.
15. Aguggini G, Berti F, Clement MG, Magni F, Rossoni G. The ginkolide BN 52021 antagonizes the increase in pulmonary pressure induced by platelet activating factor in pig: a link to $TXA_2$ generation. In: Braquet P, ed. *Ginkolides—Chemistry, Biology, Pharmacology and Clinical Perspectives. Vol 2.* Science Publishers, JR Prous, 1989.
16. Persson MG, Kalzén H, Gustafsson LE. Oxygen or low concentrations of nitric oxide reverse pulmonary vasoconstriction induced by nitric oxide synthesis inhibition in rabbits. *Acta Physiol Scand* 1994; **150**: 405–411.
17. Kirkebøen KA, Naess PA, Offstad J, Ilebekk A. Effects of regional inhibition of nitric oxide synthesis in intact porcine hearts. *Am J Physiol* 1994; **266**(Heart Circ Physiol 35): H1516–H1527.
18. Christie MI, Lewis MJ. Vascular smooth muscle sensitivity to endothelium-derived relaxing factor is different in different arteries. *Br J Pharmacol* 1988; **95**: 630–636.
19. Christie MI, Griffith TM, Lewis MJ. A comparison of basal and agonist-stimulated release of endothelium-derived relaxing factor from different arteries. *Br J Pharmacol* 1989; **98**: 397–406.
20. White DW, Martin W. Differential control and calcium-dependence of production of endothelium-derived factor and prostacyclin by pig aortic endothelial cells. *Br J Pharmacol* 1989; **97**: 683–690.
21. Förstermann U, Alheid U, Hohlfeld J. Endothelial production of prostacyclin and endothelium-derived relaxing factor is controlled by separate mechanisms. In: Rubanyi GM, Vanhoutte PM, eds. *Endothelium-Derived Vasoactive Factors, Vol 1.* Basel: Karger, 1990; 291–302.
22. Takekoshi K, Kasai K, Suzuki Y, Sekiguchi Y, Banba N, Nakamura T, Shimoda SI. Effect of $N^G$-nitro-L-arginine on shock induced by endotoxin and by platelet activating factor in dogs. *Eur J Pharmacol* 1993; **250**: 465–467.
23. Albertini M, Clement MG. In pigs, inhaled nitric oxide (NO) counterbalances PAF-induced pulmonary hypertension. *Prost Leuk Ess Fatty Acids* 1994; **51(5)**: 357–362.
24. Filep JG, Földes-Filep É. Modulation by nitric oxide of platelet-activating factor-induced albumine extravasation in the conscious rat. *Br J Pharmacol* 1993; **110**: 1347–1352.
25. May GR, Crook P, Moore PK, Page CP. The role of nitric oxide as an endogenous regulator of platelet and neutrophil activation within the pulmonary circulation of the rabbit. *Br J Pharmacol* 1991; **102**: 759–763.
26. Belvisi MG, Stretton CD, Yacoub M, Barnes PG. Nitric oxide is the endogenous neurotransmitter of bronchodilator nerves in humans. *Eur J Pharmacol* 1992; **210**: 221–222.
27. Tucker JF, Brane SR, Charalambons L, Hobbs AJ, Gibson A. L-$N^G$-nitro-arginine inhibits non-adrenergic, non cholinergic relaxation of guinea pig isolated tracheal smooth muscle. *Br J Pharmacol* 1990; **100**: 663.

**Received 14 October 1994; accepted in revised form 21 December 1994**
